# Transcriptome profiling of human hepatocytes treated with Aroclor 1254 reveals transcription factor regulatory networks and clusters of regulated genes

**DOI:** 10.1186/1471-2164-7-217

**Published:** 2006-08-26

**Authors:** Susanne Reymann, Jürgen Borlak

**Affiliations:** 1 Fraunhofer Institute of Toxicology and Experimental Medicine (Fh-ITEM), Center for Drug Research and Medical Biotechnology, Nikolai-Fuchs-Str. 1, 30625 Hannover, Germany; 2 Center of Pharmacology and Toxicology, Medical School of Hannover, Carl-Neuberg-Str. 1, 30625 Hannover, Germany

## Abstract

**Background:**

Aroclor 1254 is a well-known hepatotoxin and consists of a complex mixture of polychlorinated biphenyls (PCBs), some of which have the ability to activate the aryl hydrocarbon receptor (AhR) and other transcription factors (TFs). Altered transcription factor expression enables activation of promoters of many genes, thereby inducing a regulatory gene network. In the past, computational approaches were not applied to understand the combinatorial interplay of TFs acting in concert after treatment of human hepatocyte cultures with Aroclor 1254. We were particularly interested in interrogating promoters for transcription factor binding sites of regulated genes.

**Results:**

Here, we present a framework for studying a gene regulatory network and the large-scale regulation of transcription on the level of chromatin structure. For that purpose, we employed cDNA and oligomicroarrays to investigate transcript signatures in human hepatocyte cultures treated with Aroclor 1254 and found 910 genes to be regulated, 52 of which code for TFs and 47 of which are involved in cell cycle and apoptosis. We identified regulatory elements proximal to AhR binding sites, and this included recognition sites for the transcription factors ETS, SP1, CREB, EGR, NF-kB, NKXH, and ZBP. Notably, ECAT and TBP binding sites were identified for Aroclor 1254-induced and E2F, MAZ, HOX, and WHZ for Aroclor 1254-repressed genes. We further examined the chromosomal distribution of regulated genes and observed a statistically significant high number of gene pairs within a distance of 200 kb. Genes regulated by Aroclor 1254, are much closer located to each other than genes distributed randomly all over the genome. 37 regulated gene pairs are even found to be directly neighbored. Within these directly neighbored gene pairs, not all genes were *bona fide *targets for AhR (primary effect). Upon further analyses many were targets for other transcription factors whose expression was regulated by Aroclor 1254 (secondary effect).

**Conclusion:**

We observed coordinate events in transcript regulation upon treatment of human hepatocytes with Aroclor 1254 and identified a regulatory gene network of different TFs acting in concert. We determined molecular rules for transcriptional regulation to explain, in part, the pleiotropic effect seen in animals and humans upon exposure to Aroclor 1254.

## Background

Transcription factors (TFs) play a decisive role in regulatory gene networks. By interacting with specific DNA-binding elements (TF binding sites) transcriptional activation of targeted genes can be initiated. The expression and/or activity of TFs may be regulated in a cell type- or tissue-specific or in a cell cycle-dependent manner and can be changed by different environmental influences [1]. Some TFs regulate their own transcription in a feed-back loop [2].

A well-understood ligand-activated nuclear transcription factor is aryl hydrocarbon receptor (AhR) [3]. Prior to activation through a variety of toxins such as 2,3,7,8-tetrachlorodibenzo-p-dioxin (TCDD), polycyclic aromatic hydrocarbons, combustion products, and numerous phytochemicals including flavonoids and indole-3-carbinol (I3C), AhR is found in the cytosol in association with heat shock protein HSP90 and HSP90 accessory proteins. Ligand binding releases these proteins from their complex and promotes nuclear translocation. In the nucleus, the ligand-activated AhR forms a heterodimeric complex with aryl hydrocarbon nuclear translocator (ARNT) [4]. The complex binds to aryl hydrocarbon response elements (AhRE) – also known as xenobiotic response elements (XRE) and dioxin response elements (DRE) -, which function as cis-acting enhancers in the regulatory domains of targeted genes, some of which are collectively known as the AhR gene battery [5]. Negative regulation of gene expression through AhR signaling is also known [6], but the mechanisms by which different genes are either induced or repressed after binding of activated AhR to cognate recognition sites in gene promoters remains uncertain. This is of particular importance, as ligands of AhR elicit a broad spectrum of biochemical and toxicological effects including teratogenesis, immunosuppression due to thymic involution, and tumor promotion [7].

The importance of AhR in cell cycle regulation and apoptosis has, however, only just begun to be realized [5], and there is evidence for AhR to be directly involved in the transcriptional regulation of genes participating in cell cycle regulation [8].

The metabolism of endogenous and exogenous substrates by P450 enzymes (two of which are members of the AhR gene battery) has been shown to cause reactive oxygenated metabolite (ROM)-mediated oxidative stress. Oxidative stress is a major signal in triggering apoptosis; however, the precise mechanism, or molecules, determining the cell's decision for either apoptosis or continuation of the cell cycle remains to be elucidated.

Furthermore, the importance of the chromosomal localization of genes with respect to transcriptional regulation has also just begun to be realized. Many co-expressed, non-homologous genes are known to be clustered on chromosomes, implying their co-regulation. The positional clustering of co-expressed genes is common in prokaryotes (operons) and was recently described for *Saccharomyces cerevisiae *[9], *Caenorabditis elegans *[10] and *Drosophila melanogaster *[11,12]. Throughout the human genome, it is often supposed that genes are randomly distributed, except for tandem duplicates. However, chromosomal domains of highly expressed genes have recently been revealed in the human genome as well [13,14].

Here, we used high-density oligonucleotide and cDNA microarray technology to analyze transcript signatures of Aroclor 1254-treated human hepatocytes. For the assignment of biological processes involving the Aroclor 1254-regulated genes, the GOFFA library was used, which is part of the public toxicogenomics software for microarray data management and analysis, e.g. ArrayTrack [15]. This library provides gene ontology information using the standard vocabulary (terminology) of the Gene Ontology Consortium. Specifically, we searched for novel transcription factors for an improved understanding of the regulatory gene network modulated by Aroclor 1254. Next to an identification of AhR binding sites in regulated genes, we searched for additional TFs binding sites as to determine combinatorial interactions and to determine the reason for the level of genes being either induced or repressed. We also examined the chromosomal distribution of Aroclor 1254-regulated genes to test for our hypothesis of transcriptional regulation of co-localized genes on the basis of regulation of neighborhood genes which are not *bona fide *targets of AhR but are targets of other regulated TFs. Therefore we report molecular rules for transcriptional regulation of targeted genes to explain in part the pleiotropic effect observed in animals and humans upon exposure to Aroclor 1254.

## Results

### Analysis of high-density oligonucleotide microarray data

**Figure 1:**
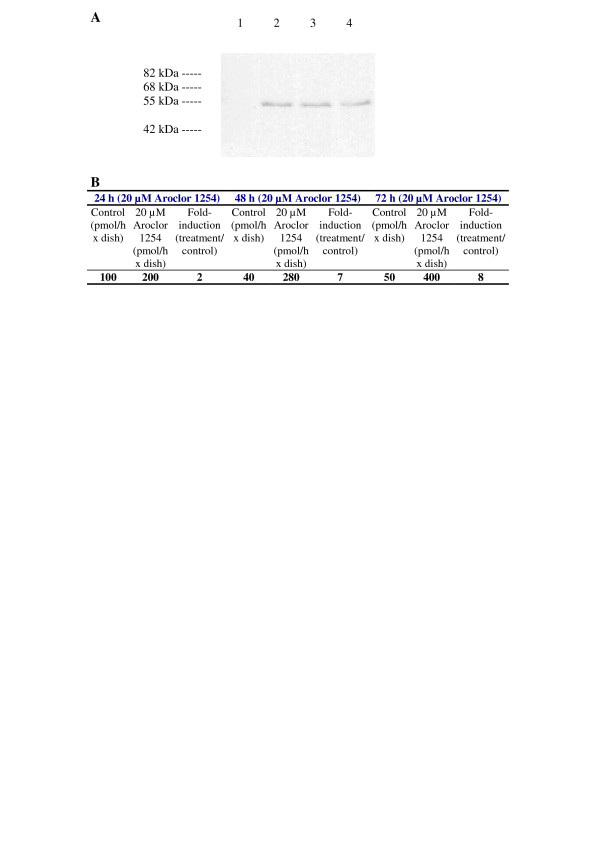
**Confirmation of AhR activation by components of Aroclor 1254 by CYP1A1 western blotting experiments and by determining EROD enzyme activity**. A: Western blot using an antibody against CYP1A1. Protein expression of CYP1A1 in cultures of human hepatocytes treated with three different concentrations of Aroclor 1254 (10 μM, 20 μM, and 50 μM) for 72 h, respectively. Lane 1: control, untreated; lane 2: 10 μM Aroclor 1254; lane 3: 20 μM Aroclor 1254; lane 4: 50 μM Aroclor 1254. B: EROD-enzyme activity. Enzyme activity of CYP1A1 in cultures of human hepatocytes treated with 20 μM Aroclor 1254 for 24 h, 48 h and 72 h, respectively. The de-ethylation product resorufin was determined fluorometrically. Results are mean and SD of three dishes.

We used RNA from control and Aroclor 1254-treated hepatocyte cultures oligonucleotide microarrays. The complete data have been deposited in NCBIs Gene Expression Omnibus (GEO) [16] and are accessible through GEO Series accession number GSE5213. The experiment revealed that 910 genes were regulated by Aroclor 1254. Aroclor 1254 treatment resulted in increased expression of 465 genes (at least two-fold), and repressed expression of 445 genes (by at least 50%), as compared to controls. AhR activation by components of Aroclor 1254 was also confirmed by gene expression profiling with cDNA arrays. Here, the expression ratio of CYP1A1 was 31.3, the expression ratio of CYP1A2 was 11.9 and the expression ratio of UGT1A1 was 2.9 as compared to controls. Further, AhR activation by components of Aroclor 1254 was confirmed by CYP1A1 Western blotting experiments and by determining EROD enzyme activity (Figure 1). We found CYP1A1 protein expression and enzyme activity to be clearly induced in cultures of human hepatocytes after incubation with Aroclor 1254. These data provide conclusive evidence for activation of AhR in cultures of human hepatocytes treated with Aroclor 1254.

### Cellular roles of Aroclor 1254-regulated genes

**Figure 2:**
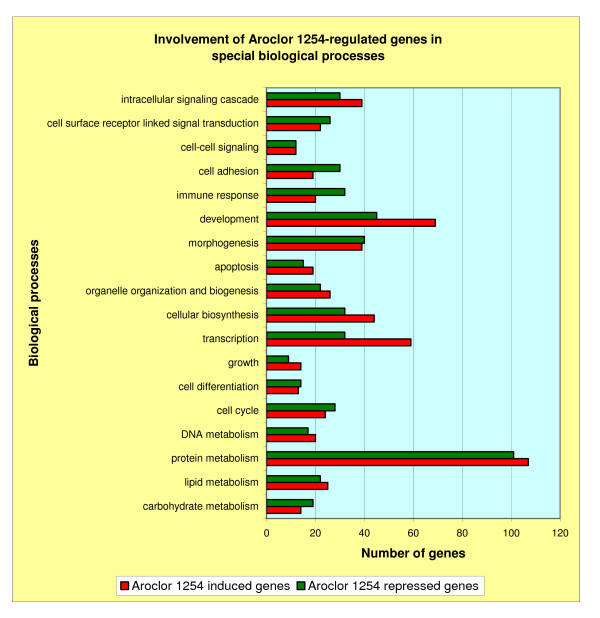
**Comparison of Aroclor 1254-induced genes and Aroclor 1254-repressed genes in respect of their involvement in different biological processes**. The different biological processes are given on the ordinate the number of genes involved in these processes is given on the abscissa. Red bars: Aroclor 1254-induced genes; green bars: Aroclor 1254-repressed genes.

To assign the biological processes involving the Aroclor 1254-regulated genes, we used the GOFFA library, which is a part of the public toxicogenomics software for microarray data management and analysis ArrayTrack [15]. This library provides gene ontology information, using the standard vocabulary (terminology) of the Gene Ontology Consortium. We focused only on some particular biological processes, as depicted in Figure 2, and separately assigned the number of Aroclor 1254-induced and Aroclor 1254-repressed genes which are involved in these processes. Corresponding to the number of biological processes a particular gene is involved in, this gene can appear in different processes. Comparison of induced and repressed genes' involvement in the different biological processes (Figures 2) demonstrates that there was preponderance for Aroclor 1254-induced genes to be involved more often in the gene/protein expression machinery, in the intracellular signal cascade and in development, whereas repressed genes were more often involved in immune response and cell adhesion. This result reflects the reaction of the cells to the changes of environmental influences, here especially the influence of the mixture of xenobiotics (Aroclor 1254).

### Aroclor 1254-regulated expression of transcription factors and genes involved in regulation of cell cycle and apoptosis

We focused on genes coding for transcription factors in order to elucidate the regulatory gene network induced by treatment of human hepatocytes with Aroclor 1254. We further focused on genes coding for regulators of cell cycle and apoptosis (for review of the role of AhR in cell cycle control and apoptosis see [5]), even though only limited information on the specific role of AhR is available [8].

28 out of 465 genes which are induced by Aroclor 1254 treatment are transcription factors (one of which does not bind directly to the DNA, but rather is a co-regulator of transcription). Many of these transcription factors are stress-induced and transcriptional regulate genes which are involved in apoptosis, cell cycle, development, morphogenesis, and oncogenesis as described in Additional file 2a. 22 out of 465 genes which are induced by Aroclor 1254 treatment are especially involved in regulation of cell cycle and apoptosis. Additional file 2a depicts their Entrez Gene identifier and their signal ratio and furthermore the classification and function of these transcription factors. We also identified 24 out of 445 genes which are repressed as transcription factors by Aroclor 1254 treatment (five of which do not bind directly to the DNA, they are rather co-regulators of transcription) and 25 genes which are especially involved in regulation of cell cycle and apoptosis. They are listed in Additional file 2b, including their Entrez Gene identifier and signal ratio. Moreover, this Table depicts the classification and function of the transcription factors. Many of these transcription factors are transcriptional repressors and are involved in the regulation of genes which play a role in cell cycle, development and oncogenesis.

### Promoter sequence analysis of Aroclor 1254-regulated transcription factors and of genes involved in regulation of cell cycle and apoptosis

After identification of the Aroclor 1254-regulated transcription factors and genes involved in regulation of cell cycle and apoptosis, we extracted their promoters from public databases and analyzed them in respect of AhR binding sites. For this purpose, we used a matrix which we had recently constructed (TRANSFAC accession no. M00778; id V$AHR_Q5) [17]. As controls, we used promoters of randomly selected genes from the microarray which were not influenced by Aroclor 1254-treatment (relative expression value = 1.0). Two different cut-off values, q_cut-off _= 0.96 and q_cut-off _= 0.98, were chosen for computational analysis. We applied two different cut-offs in order to test for an increase of the fold occurrences by using the more stringent cut-off value (q_cut-off _= 0.98). This would be a conformation for the specificity of the matrix used (see below and Table 1). The numbers of AhR binding sites in the promoters of Aroclor 1254-regulated transcription factors are given in Additional file 2a and Additional file 2b, respectively, whereas the numbers of AhR binding sites in promoters of unregulated genes are listed in Additional file 3.

The calculated fold occurrences of AhR sites in the analyzed gene promoters are given in Table 1. They describe the number of AhR binding sites detected as a ratio with regard to the number of gene promoters analyzed. Here, the fold occurrence of the unregulated gene promoters is set to 1. Using the lower cut-off value of 0.96, the fold occurrences of the AhR sites of the analyzed promoters of one group in total are clearly increased in comparison to the fold occurrences of the AhR sites of the control gene promoters in total, with the exception of Aroclor 1254-induced genes involved in apoptosis. However, using the more stringent matrix similarity cut-off value of 0.98, the fold occurrences of the AhR sites in the promoters of all Aroclor 1254-regulated genes including genes involved in apoptosis is clearly increased as compared to promoters of unregulated genes. This result once more confirms the specificity of the AhR matrix used and is an indication for a direct regulation by AhR of many of the genes analyzed.

In order to prove whether AREs/EpREs (antioxidant responsive elements/electrophile responsive elements), which belong to an AhR-independent chemoprotective response system, also play a role in regulation of the analyzed promoters, we constructed an ARE weight matrix using functional ARE-DNA sequences [18]. Further, we applied this ARE matrix to analyze all extracted promoters of Aroclor 1254-regulated genes (Aroclor 1254-regulated transcription factors and genes involved in regulation of cell cycle and apoptosis). No ARE could be detected (data not shown). As positive control for the matrix we used the rat GST-Ya gene promoter, which contains several ARE recognition sites (data not shown).

### Common frameworks in the promoters of Aroclor 1254-regulated genes

We used the Genomatix software FrameWorker to extract a common framework consisting of different transcription factor recognition sequences proximal to AhR. This tool is designed for the comparative analysis of promoter sequences. FrameWorker returns the most complex models that are common to the input sequences. These are all elements that occur in the same order and in a certain distance range in all (or a subset of) the input sequences. Here, we aimed to identify further transcription factor binding sites next to that one of the AhR in order to determine rules for AhR-mediated transcriptional regulation in response to the treatment of human hepatocytes with Aroclor 1254. We further whished to identify differences between Aroclor 1254-induced and -repressed promoters. Because positive as well as negative regulation of gene expression through AhR signaling is known, but the mechanisms by which different genes are either induced or repressed after binding of activated AhR to cognate recognition sites in gene promoters remains uncertain.

For this purpose we analysed Aroclor 1254-induced and repressed promoters separately. The results are shown in Table 2. Besides at least one AhR binding site, all Aroclor 1254-regulated gene promoters (induced and repressed) were defined by ETS and SP1 binding sites. All Aroclor 1254-repressed gene promoters additionally contained E2F binding sites. Furthermore, at least 90% of all analysed Aroclor 1254-regulated gene promoters, contained CREB, EGR, ZBP, NF-kappaB, and NKXH binding sites. Notably, 90% of the Aroclor 1254-induced gene promoters were positive for ECAT and TBP, whereas 90% of the Aroclor 1254-repressed genes contained MAZ, HOX, and WHZ binding sites. Thus, Aroclor 1254-induced and -repressed genes could be distinguished on the basis of combinatory rules for transcription factor recognition sequences. As control we analysed the promoters of genes which were not regulated by Aroclor 1254 at all (see Additional file 3) by using the FrameWorker. 90 % of these promoters were positive for binding sites of only one single transcription factor: ETS. No model with at least 2 elements was found (data not shown).

### Chromosomal localization of Aroclor 1254-regulated genes

For 448 out of 465 up-regulated genes and for 434 out of 445 repressed genes the chromosomal localization could be determined. The relative distribution of Aroclor 1254-regulated genes on the different chromosomes showed no marked under- or overrepresentation for particular chromosomes as compared to the distribution of all genes of the genome mapped so far (Additional file 1: Here, chromosomal distribution data for all genes were plotted against Aroclor 1254-regulated genes) (Distribution of genes on the Y chromosome could not be studied as data from male and female donors were pooled).

**Figure 3:**
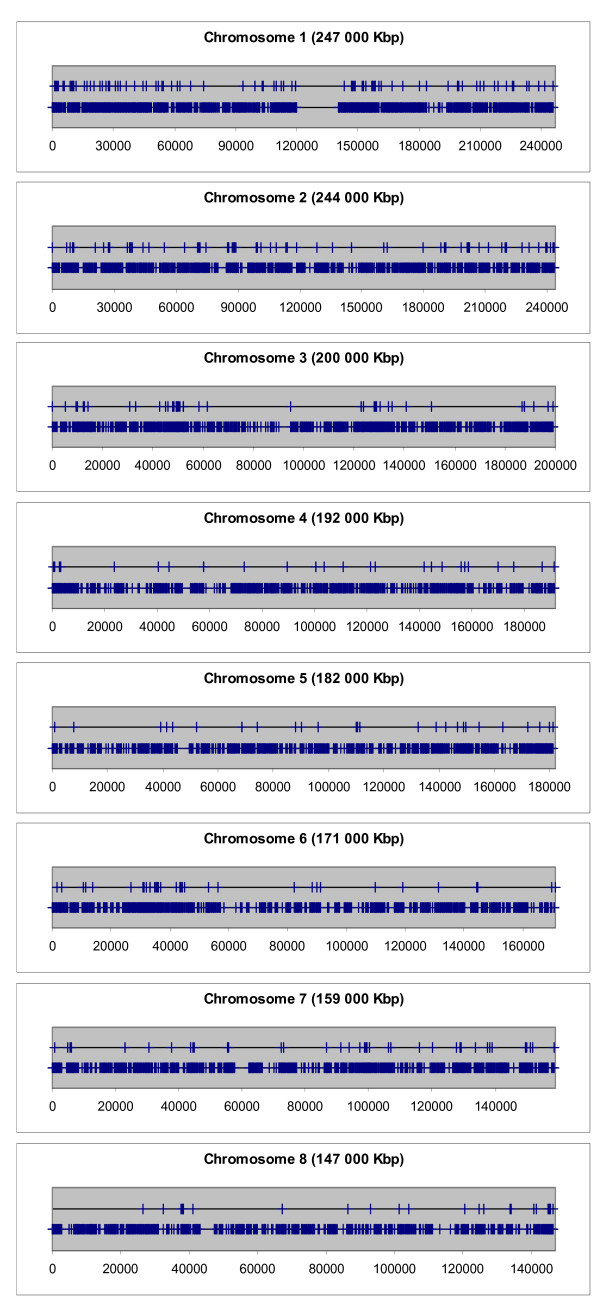
**Distribution of Aroclor 1254-regulated genes on the chromosomes 1–8**. The positions of the transcription start sites (in kbp) of the Aroclor 1254-regulated genes are marked on the upper horizontal black line as vertical blue bars, respectively. For comparison, the positions of the transcription start sites of all genes mapped so far are marked on the lower horizontal black line as vertical blue bars, respectively.

**Figure 4:**
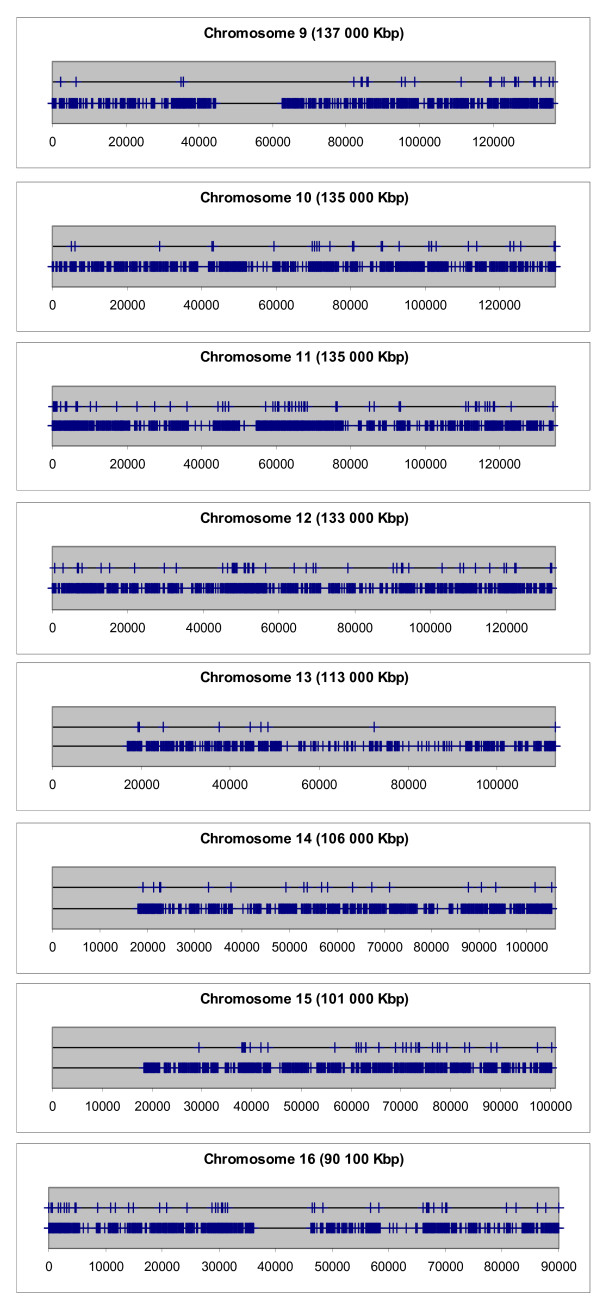
**Distribution of Aroclor 1254-regulated genes on the chromosomes 9–16**. The positions of the transcription start sites (in kbp) of the Aroclor 1254-regulated genes are marked on the upper horizontal black line as vertical blue bars, respectively. For comparison, the positions of the transcription start sites of all genes mapped so far are marked on the lower horizontal black line as vertical blue bars, respectively.

**Figure 5:**
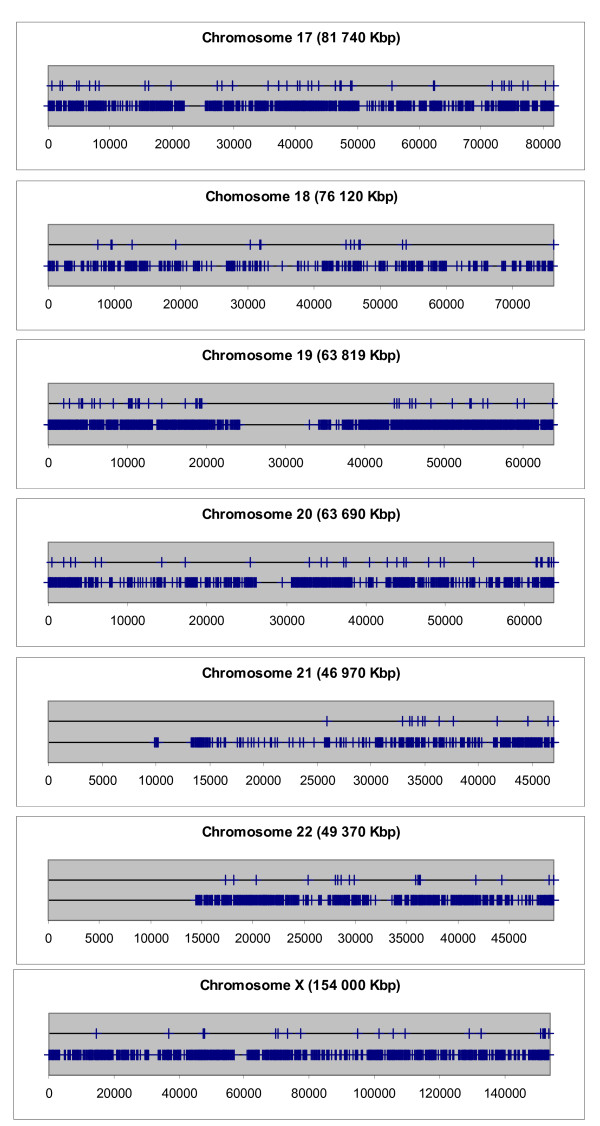
**Distribution of Aroclor 1254-regulated genes on the chromosomes 17–22 and on the X chromosome**. The positions of the transcription start sites (in kbp) of the Aroclor 1254-regulated genes are marked on the upper horizontal black line as vertical blue bars, respectively. For comparison, the positions of the transcription start sites of all genes mapped so far are marked on the lower horizontal black line as vertical blue bars, respectively.

Next, the distribution of Aroclor 1254-regulated genes on the single chromosomes was delineated graphically. The distribution of Aroclor 1254-regulated genes on the chromosomes in comparison with the distribution of all genes mapped so far is shown in Figures 3, 4 and 5. These graphs provide the visual impression that many Aroclor 1254-regulated genes are co-localized in proximal neighborhood and they seem to form clusters within chromosomal regions. However, it is known that genes in general are not uniformly distributed in the genome, as shown in Figures 3, 4 and 5. In order to answer the question as to whether Aroclor 1254-regulated genes are statistically significant co-localized along the chromosomes, we calculated specifically the probability by which Aroclor 1254-regulated genes occur within DNA windows of different sizes as compared to the probability of all known mapped 27,400 genes (RefSeq transcripts) of the human genome to occur within the same DNA windows. The results of this analysis are depicted in Table 3. The numbers of observed pairs of Aroclor 1254-regulated genes in the different window sizes used and the mean of the numbers of pairs of genes stemming from the 100 random lists in the different window sizes used are listed in Table 3. In this table also the standard deviations of the mean values of the random lists are given, as well as the significances (*p value*), which express the possibility by which such a result would appear by chance. The significance was calculated by using the binominal distribution. Table 3 depicts the over-representation of co-localized gene pairs within the window sizes >0–50, >50–100 and >100–200 kbp. These results are shown to be statistically highly significant (in Table 3). Thus, genes which were regulated by Aroclor 1254 are much closer located to each other than genes distributed randomly all over the genome. RefSeq transcripts which possess the same start site of transcription and thus being alternatively spliced transcripts are included only one time in the window size of 50 kbp. The other was skipped from the analysis. Next, we analysed whether the identified Aroclor 1254-regulated gene pairs are directly neighbored (Note, 'directly neighbored gene pair' means simply two genes adjacent to each other, in contrast to 'gene pair', which is localized within a set window). Indeed, we found 37 gene pairs to be directly neighbored on the chromosomes. The distances of the transcription start sites of these directly neighbored gene pairs range from 3 kb to 160 kb. Strikingly, the size of chromatin domains, which are the units of transcriptional competences, varies from 5 kb to 200 kb [19]. A detailed description of the 37 directly neighbored gene pairs is shown in Additional file 4.

In order to explore whether Aroclor 1254-regulated transcription factors (see Additional file 2) also play a role in the indirect regulation of genes being directly neighbored, we used the corresponding available weight matrices from the database TRANSFAC^® ^Professional rel. 10.1 for the analysis of all extracted promoters [exclusively, promoters of genes, which are RefSeq annotated were extracted. The beginning of the first exon, which also comprises the 5'UTR was considered to be a tentative TSS (transcription start site)] of Aroclor 1254-regulated directly neighbored genes [ATF3 (TRANSFAC^® ^acc. number: M00513), v-Jun (M00036), HOXB7 (M00998), SP2 (M00933), PBX1 (M00998, M01017), MAF/NRF1 (M00983), NF-kappaB (M00208, M00774, M00054, M00194), PPARG2 (M00512, M00515), SOX18 (M01014) and BHLHB2 (M00997, M01034)]. Unfortunately, matrices of the other Aroclor 1254-regulated transcription factors are not yet available. The result of this analysis is shown in Table 4. In 57 out of 73 analysed promoter sequences of the directly neighbored genes binding sites of either AhR or/and NF-kappaB or/and ATF3 or/and SP2 or/and PPARG2 or/and RFXANK or/and NRF1 or/and BHLHB2 were identified. In only 16 out of 73 analysed promoter sequences no transcription factor binding site could be detected, neither an AhR binding site nor a binding site of an Aroclor 1254-regulated transcription factor, whose matrice was available in the database TRANSFAC^® ^Professional rel. 10.1. Notably, these genes might be targets of other Aroclor 1254-regulated transcription factors whose matrices are not yet available. Thus, once a chromatin domain of about 200 kbp is opened for the primary targets of Aroclor 1254 (e.g. AhR) the promoters of genes located within this domain might be accessible also for secondary targets of Aroclor 1254 (e.g. transcription factors other than AhR whose expression is influenced by Aroclor 1254; see Additional file 2).

## Discussion

AhR-mediated gene regulation has been defined by a sequence of events by which the ligand-activated AhR translocates into the nucleus and binds to AhR-DNA binding sites in the promoters, thereby altering gene expression controlled by these promoters [3]. This mode of action limits the regulation of genes by the ligands of the AhR, including polyhalogenated and polycyclic aromatic hydrocarbons (PAHs), to those containing AhR binding sites.

It has often been described in the literature, however, that xenobiotics bound to the AhR cause a large number of apparently unrelated biological and toxic effects [20], suggesting that there exist other mechanisms to cause the observed responses without acting through AhR binding sites in the promoters of the corresponding genes.

In this study, we performed computational analyses in order to elucidate the network of transcription factors and their target genes in response to AhR activation or activation of as yet unknown factors by Aroclor 1254 in cultures of human hepatocytes.

**Figure 6:**
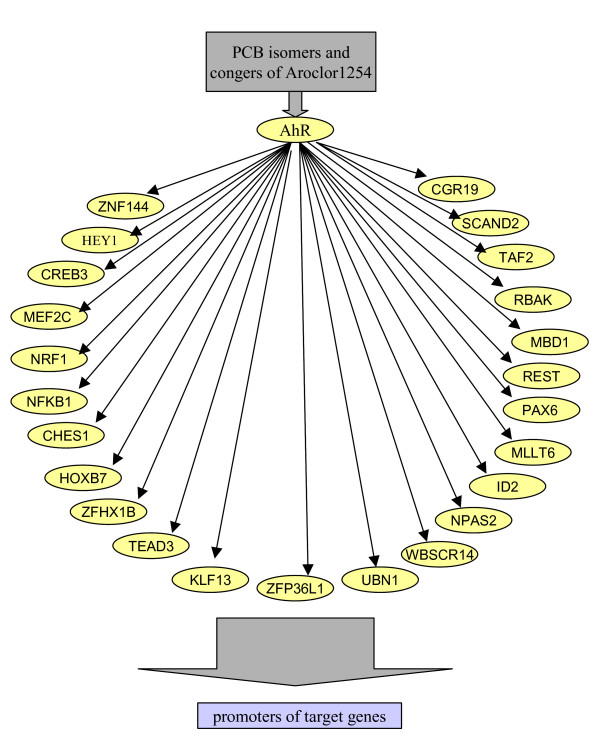
**Scheme of proposed network of transcription factors involved in regulation of gene expression under direct or indirect influence by the AhR**. This network of transcription factors may explain the regulation of several genes that are not direct targets of AhR. Their regulation can be mediated through other TFs whose expression is regulated directly by AhR.

Activation and repression of transcription factors and the ensuing changes in gene regulation seem to be hallmarks of the molecular mechanisms of Aroclor 1254. Many of them are candidates for being regulated directly by AhR, through its binding to the corresponding *in silico*-identified DNA binding sites in their promoters. This proposed network of transcription factors involved in regulation of gene expression under direct or indirect influence by the AhR is depicted in Figure 6.

In spite of the considerably higher fold occurrence of AhR binding sites in the whole of Aroclor 1254-regulated gene promoters analyzed as compared to non-influenced gene promoters, many of them possess no AhR binding sites at all. These genes might be regulated by Aroclor 1254 for various reasons: 1) The changes in the expression of these genes might be secondary effects, maybe through the influence of transcription factors the expression of which is regulated by AhR directly. Thus, the directly regulated transcription factors seem to be the basis for the regulatory gene network induced by Aroclor 1254, through the ensuing regulation of their target genes. 2) The changes in the expression of these genes might also be direct effects through an as yet unknown influence of Aroclor 1254 on other transcription factors. Parallel activity of activated TFs would be plausible causes for the pleiotropic effects seen in animals upon treatment with PCBs. 3) Furthermore, it is known that functional AhR sites may be situated in the promoter regions of the AhR target genes, in their introns as well as in far upstream and downstream non-coding regions around the genes. In 2004, however, we could demonstrate that within the window of -1000 bp upstream from the start of transcription AhR sites could be observed with the highest frequency in AhR-regulated genes [17]. For that purpose, we decided to analyze exclusively these regions of the promoters. Nevertheless, we cannot exclude the possibility that we lose information by this means. 4) These genes might also be regulated through co-localization to directly regulated genes within a chromatin domain (detailed discussion see below).

In spite of using an AhR matrix, the quality of which has been demonstrated to be high [17], it must be mentioned that the *in silico *identification of transcription factor binding sites in DNA sequences is not a sufficient proof for binding of the corresponding factor *in vivo*. Moreover, in some cases binding of transcription factors *in vivo *occurs in promoters of genes whose expression is not altered [21].

Despite this limitation the computational analysis of microarray data reveal novel insight into the regulation of networks of TFs acting in concert.

Indeed, one of the Aroclor 1254-induced transcription factors is NF-kappaB (p105). NF-kappaB is a key regulator of immune, inflammatory, and acute phase responses and is also involved in the control of cell proliferation, apoptosis, and oncogenesis. NF-kappaB is known to control many physiological functions adversely affected by AhR ligands. Remarkable progress has been made in understanding the signal transduction pathways that lead to activation of NF-kappaB factors and consequent induction of gene expression [22]. In this study, expression of NF-kappaB was induced about 11-fold by Aroclor 1254. We identified a single AhR binding site in its gene promoter. Therefore, the transcription might be induced through binding of activated AhR directly. The existing literature provides no information regarding activation of NF-kappaB gene expression by AhR. However, a recent study showed physical interaction and mutual functional repression between AhR and NF-kappaB [23]. Protein-protein interactions between TFs are a further important mode of action to increase their regulatory repertoire.

In the past few years, the importance of combinatorial regulation of transcription by networks of transcription factors has begun to be realized. In this study, we identified several transcription factor binding sites occurring proximal to AhR in the Aroclor 1254-regulated promoters, amongst them NF-kappaB. They belong to a common framework of transcription factors acting in concert in response to Aroclor 1254. Thus, also in transcriptional regulation, AhR and NF-kappaB seem to work together. Moreover, CREB and SP1 were found to be part of this framework. The CREB transcription factor is a member of the leucine zipper family. This protein binds as a homodimer to the cAMP-responsive element, an octameric palindrome. The protein is phosphorylated by several protein kinases and induces transcription of genes in response to stimulation of the cAMP pathway. In this study, CREB was shown to be induced by Aroclor 1254, maybe directly through binding of AhR to the *in silico*-identified AhR binding sites in its promoter. The transcription factor Sp1, which was not determined on the Aroclor 1254 microarray, is a DNA-binding protein which interacts with a variety of gene promoters containing GC-box elements and is known to physically and functionally interact with AhR in promoters of several genes in which SP1 plays an important role in basal gene expression [24]. Moreover, a functional synergism of SP1 and the nuclear factor Y (ECAT) in transcriptional stimulation of the rat pyruvate kinase M (PKM) gene could recently be demonstrated [25]. SP1 and NF-Y (ECAT) stimulate transcription of the PKM gene via their interactions. Here, we found SP1 binding sites in 100 % of Aroclor 1254-induced as well as in 100 % of repressed gene promoters proximal to AhR binding sites, but we found ECAT binding sites exclusively in 90 % of the induced gene promoters and not in repressed promoters. Therefore, the induction of AhR-regulated transcription might also be the result of the interplay between SP1 and ECAT/NF-Y.

Furthermore, a recent study demonstrated that AhR forms a complex and synergizes with Retinoblastoma Protein (RB) to repress E2F-dependent transcription and cell cycle progression [26]. Again, a very recent study demonstrated that AhR-mediated gene repression requires combinatorial interactions at E2F-reponsive promoters, leading to the repression of E2F-dependent, S phase-specific genes [27]. By analogy, we showed E2F binding sites to be part of a common framework of all Aroclor 1254-repressed gene promoters analyzed.

ATF3, here found to be induced by Aroclor 1254, is a transcription factor that represses transcription from promoters with ATF sites through stabilization of the binding of inhibitory co-factors at the promoter [28]. ATF3deltaZip, here found to be repressed by Aroclor 1254, is an alternatively spliced form of ATF3 that encodes a truncated ATF3 which does not bind DNA, stimulates transcription and antagonizes the action of ATF3 [29]. Antagonism of both these splice forms is also reflected in the Aroclor 1254 reaction resulting from our microarray experiments, in which ATF3 is induced whereas ATFdeltaZip is reduced by Aroclor 1254 (no AhR binding site could be identified in its promoter, suggesting an indirect effect).

Another transcription factor, the expression of which was induced about three-fold by Aroclor 1254, was the peroxisome proliferator-activated receptor gamma (PPARγ). This is one example of a soluble intracellular receptor, which is important in mediating toxic responses and is associated with hepatocarcinogenesis in rodents [29].

Concerning Aroclor 1254-regulated genes involved in the regulation of cell cycle and apoptosis, some of them are remarkable and will be discussed. In our microarray analysis, the cyclin-dependent kinase 9 (CDK9 or Sprouty2) was induced 3.2-fold by Aroclor 1254. Sprouty2 is known to attenuate epidermal growth factor receptor ubiquitylation and endocytosis, and consequently enhances RAS/ERK signaling [30]. Various intracellular signaling cascades are altered in tumor cells. Among these, the receptor tyrosine kinase RAS/ERK signaling pathway was found to be constitutively active in a significant percentage of human tumors (for review see [31]). Furthermore, the cell cycle inhibitory protein p21 (Cip1) was found to be induced 5.5-fold by Aroclor 1254. The carcinogenic polycyclic aromatic hydrocarbon benzo [a]pyrene, which is a high-affinity AhR ligand, is known to cause growth arrest in 3T3 fibroblasts during the G1 phase of the cell cycle [32]. We identified a potential AhR binding site in the Cip1 promoter; hence, the induction of Cip1 by Aroclor 1254 could be a direct effect of AhR. Cdc25A, a phosphatase essential for G1-S transition, for cell cycle progression, which is known to compete against Cip1, was shown here to be reduced by 50% upon Aroclor 1254-treatment.

To gain a better understanding of the expression of individual genes in response to Aroclor 1254, it was useful to analyze the corresponding promoter sequences for regulatory modules, especially transcription factor binding sites, being responsible for quantitative changes in the expression. This resulted in models of transcriptional regulation, either induction or repression, controlled by a special network of regulatory modules. And some facts known from the literature for single genes could be confirmed here *in silico *for many genes (see above).

However, mapping the Aroclor 1254-induced transcriptome back onto the genome was useful to get insight into the large-scale regulation of transcription. Here, we found that genes regulated by Aroclor 1254, are much closer located to each other than genes distributed randomly all over the genome, which raises the possibility of chromatin structure being involved in the large-scale regulation of transcription.

Chromatin is usually described as being divided into "open" domains where genes have the potential to be expressed through the accessibility of the corresponding gene promoters for different transcription factors, and domains of "closed" regions where gene expression is shut down. The existence of co-expressed/co-localized gene clusters is consistent with a model where large chromatin regions would change their activity (openness) status in a tissue-specific manner, allowing neighboring genes to be transcribed or shut down in a coordinated way. In this study, large chromatin regions might change their activity (openness) status in response to the influence of Aroclor 1254. This means that, besides being controlled individually, e.g. through AhR, genes may also be subject to regulation according to their location within the genome. Thus, expression of co-localized genes might reflect rules in chromatin remodeling and, as a consequence, gene transcription. Notably, we corroborated our proposal by promoter analyses of directly neighbored regulated genes. Most of the promoters included binding sites of either AhR or (a) transcription factor(s) whose expression was/were found to be regulated by Aroclor 1254 (see Additional file 2 and Table 4).

## Conclusion

**Figure 7:**
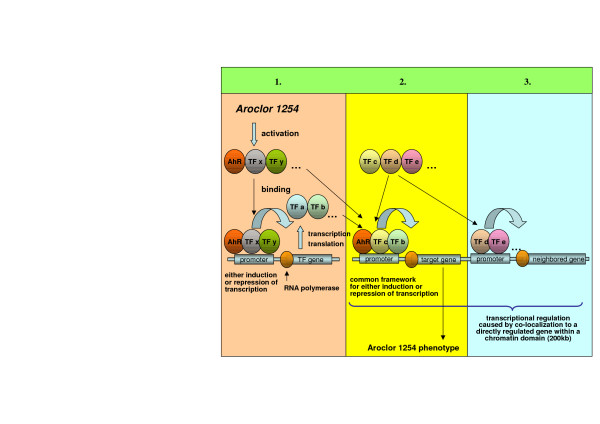
**The coordinate events which resulted in gene regulation in response to Aroclor 1254**. 1) The Aroclor 1254-activated nuclear transcription factor AhR influences the expression of different transcription factors by binding to AhR recognition sites in corresponding promoters. These transcription factors constitute the basis of a regulatory gene network that again influences the expression of different genes. Other transcription factors could also be primary targets of Aroclor 1254. 2) The Aroclor 1254-activated nuclear transcription factor AhR seems to act in concert with other transcription factors, because a common framework of the corresponding binding sites could be identified proximal to AhR in the Aroclor 1254-regulated gene promoters. The combination of co-acting transcription factors might be one reason for the level of gene expression being either induced or repressed. Other transcription factors whose sites are found in the neighborhood of AhR binding sites could well be primary targets of Aroclor 1254. 3) Chromosomal localization seems to be important in the large-scale regulation of mRNA transcripts in response to Aroclor 1254. Hence, genes in neighborhood of direct Aroclor 1254-induced transcriptional regulation (primary effect), might be co-expressed through the accessibility of their promoters for transcription factors whose transcription was altered by Aroclor 1254 (secondary effect).

Taken collectively, global gene regulation in response to Aroclor 1254 seems to be the result of different coordinate events (see Figure 7).

1: The Aroclor 1254-activated nuclear transcription factor AhR influences the expression of different transcription factors by binding to AhR recognition sites in corresponding promoters. These transcription factors constitute the basis of a regulatory gene network that again influences the expression of different genes. It has to be kept in mind here that also other transcription factors could well be primary targets of Aroclor 1254.

2: The Aroclor 1254-activated nuclear transcription factor AhR seems to act in concert with other transcription factors, because a common framework of the corresponding binding sites could be identified proximal to AhR in the Aroclor 1254-regulated gene promoters. The combination of co-acting transcription factors might be one reason for the level of gene expression being either induced or repressed. Here, it also has to be kept in mind that transcription factors whose sites are found in the neighborhood of AhR binding sites could well be primary targets of Aroclor 1254.

3: Chromosomal localization seems to be important in the large-scale regulation of mRNA transcripts in response to Aroclor 1254. Hence, genes in neighborhood of direct Aroclor 1254-induced transcriptional regulation (primary effect), might be co-expressed through the accessibility of their promoters for the transcription factors whose expression were influenced by Aroclor 1254 (secondary effect).

Our findings with AhR may well translate to a more general principle of gene transcription, and with the advent of microarray studies combined with well performed bioinformatics distinction of targeted gene transcription from co-expression of nearby genes within a chromatin domain becomes feasible.

## Methods

### Tissue and cultures

Approval was obtained from the ethics committee of the Medical School of Hanover. Human hepatocytes were isolated from surgical material. The liver specimens were stored in ice cold physiologic saline solution and immediately transported to the laboratory. Vessels visible on the cut surface were cannulated and the liver specimen was perfused with 200 ml buffer I containing 8.3 g/l NaCl, 0.5 g/l Kcl, 2.4 g/l HEPES and 0.19 g/l EGTA at pH 7.4 and 37°C. This was followed by perfusion with 200 ml of buffer II at pH 7.4 and 37°C. Buffer II consisted of 8.3 g/l NaCl, 0.5 g/l Kcl, and 2.4 g/l HEPES. Thereafter, collagenase perfusion was performed initiated with 200 ml of buffer III containing 100 μg collagenase type IV, 3.9 g/l NaCl, 0.5 g/l Kcl, 2.4 g/l HEPES and 0.7 g/l CaCl_2 _× 2 H_2_O at 37°C. The collagenase perfusate was recirculated. Flow rates ranged between 20 to 50 ml and were adapted to the size of the surgical specimen. Following perfusion, the liver capsule was carefully removed and the cells were liberated by gentle shaking of the liver into ice cold buffer IV. Buffer IV contained 9.91 g/l Hanks buffered salt without calcium and magnesium, 2.4 g/l HEPES and 2.0 g/l bovine serum albumin. The resulting cell suspension was filtered through a nylon mesh with 100 μm pore size and washed three times with buffer IV at 4°C. Viability of the hepatocytes ranged between 85% and 95% as assessed by trypan blue exclusion. On average, 1–5 × 10^9 ^cells were obtained from one isolation procedure. Primary human hepatocytes were cultured enclosed in two layers of collagen as described previously [33,34]. 1 ml collagen solution containing 1.1 mg/ml collagen was used for coating one 60 mm Petri dish (Greiner, Frickenhausen, Germany). The pH of the collagen was adjusted to 7.4 using a 10× DMEM concentrate (Biochrom, Berlin, Germany). Thirty minutes after coating the dishes, 2 × 10^6 ^hepatocytes per dish were seeded. After two hours following seeding and cell attachment, culture medium was removed along with non-adherent cells. After 24 h in culture, the medium was aspirated and a second layer of collagen was pipetted on top of the cells. After gelation of this second layer, culture medium was added. Culture medium (2 ml) was changed daily. The total volume of cell culture supernatant was 4 ml, including the hydrated collagen matrix volume of about 2 ml.

Prior to treatment with polychlorinated biphenyls, cells were allowed to recover from the isolation procedure for 4 to 5 days. Solutions of Aroclor 1254 at strength of 10 mg/ml were prepared in DMSO. These solutions were diluted as necessary with the cell culture medium to obtain doses of 20 μM Aroclor 1254. The final concentration of DMSO in the cell culture medium was 0.5% (v/v). Cell cultures receiving 0.5% DMSO with the culture medium were used as controls. After 72 h of treatment with Aroclor 1254 cells and the collagen layer were immediately frozen at -80°C.

### RNA isolation and production of copy RNA

Total RNA was isolated using QIAGEN's RNeasy total RNA isolation kit according to the manufacturer's recommendations. 10 μg of total RNA were used for the synthesis of double-stranded cDNA with SuperScript II RT. Primer extension was performed using HPLC-purified T7-(dT)_24 _(GenSet SA) as a primer. After clean-up, the double-stranded cDNA was used for synthesis of biotin-labelled cRNA (ENZO^® ^BioArray High Yield RNA Transcript Labeling Kit, Affymetrix). cRNA was purified with RNeasy spin columns from QIAGEN and cleaved into fragments of 35–200 bases by metal-induced hydrolysis.

### Microarray experiments

Oligonucleotide microarrays were hybridized and washed according to the manufacturer's recommendations as detailed in [35]. With the NimbleGen platform (25 mer) a total of 2 arrays were employed. Further, we employed 15 cDNA arrays containing 302 well-known genes with known or suspected roles in drug oxidation/detoxification, cell proliferation, tumor development, stress response, signal transduction pathways (protein kinases, cytokines), apoptosis and cell cycle arrest.

When starting with non-amplified RNA approximately 40 μg of total RNA were combined with a control RNA consisting of an *in vitro *transcribed *E. coli *genomic DNA fragment carrying a 30 nt poly(A)^+^-tail (EC17), and mRNA was isolated (Oligotex mRNA Mini Kit, Qiagen). 0.8 μg mRNA or 2 μg amplified RNA (aRNA) were diluted to 17 μl and combined with 2 μl of a second control RNA, a mixture of the three different transcripts EC7, EC20, and EC21. mRNA and aRNA were reverse transcribed by adding a mix consisting of 8 μl 5 × First Strand Buffer (GIBCO), 2 μl Primer-Mix (oligo(dT) and randomeres for mRNA or randomeres only for aRNA) (MEMOREC), 2 μl low C dNTPs (10 mM dATP, 10 mM dGTP, 10 mM dTTP; 4 mM dCTP), 2 μl FluoroLink™ Cy3/5-dCTP (amersham pharmacia biotech), 4 μl 0,1 M DTT, and 1 μl RNasin (20–40 U) (Promega). 1 μl (200 u) of SuperScriptII enzyme was added, incubated at 42°C for 30 min followed by addition of a further 1 μl of SuperScriptII enzyme and incubated under the same conditions as detailed above. 0.5 μl of RNaseH (GIBCO) was added and incubated at 37°C for 20 min to hydrolyze RNA. Cy3 and Cy5 labeled sample were combined and cleaned up using QIAquick™ (Qiagen). Eluents were concentrated in a speedVac at 45°C to obtain a volume of 10 μl. 10 μl of 2 × Hybridization Solution (MEMOREC) pre-warmed at 42°C was added. Hybridization was performed according to manufactures guidelines (MEMOREC). Briefly, cDNA-arrays were heated at 98°C in distilled water for 2 min to denature double stranded DNA and immediately mixed with 96% Ethanol. Arrays were dried by centrifugation at 500 × g and placed into a dust free cassette. 20 μl of pre hybridization buffer was heated at 98°C for 2 min, cooled to 42°C, applied to the cDNA-array and sealed with cover slip. The arrays were placed into an humidified hybridization cassette (PIQOR-HybChamb, MEMOREC) and incubated at 62°C for 30 min. The hybridization cassette was cooled to 25°C, cover slips were removed from cDNA-array and 20 μl of the labeled sample was directly applied to the cDNA-array, again sealed with a cover slip and incubated in the humidified hybridization chamber at 62°C overnight. Arrays were washed twice with washing buffer at 50°C, dried by centrifugation and stored in a dust free cassette until read out.

### Western blot analysis

Western blot analysis of human CYP monooxygenases was done as described previously [36]. In the case of CYP1A1 the monoclonal antibody mAb1A3-03 was purchased from Rubitec, USA.

### Analysis of microarray data

With both platforms applied the statistical analysis was performed using the manufacturer's software and according to its recommendations. The NimbleGen data have been normalized as follows: each set of expression values is divided by the standard deviation of that array only. The corresponding expression values were the average difference values for each target. These are calculated as the average of the signal intensities for the perfect match probe (PM) minus the mismatch probe (MM). These designs both used 10 probe pairs per target. Values that differ from the mean by more than 2.5 standard deviations are dropped from the calculation. The normalized data were then rescaled to account for between chip variations. Here, the standard deviation of the whole set of arrays were used to give us a scaling factor.

For further analysis of NimbleGen-high-density light-directed oligonucleotide microarrays, we used the ratios between the normalized intensity values for controls (non-treated human hepatocytes) and the normalized intensity values for Aroclor 1254-treated human hepatocyte cultures. A ratio of 1.0 means, that there was no change at all. At first, we sorted the genes according to their ratios. We further analyzed exclusively those genes which were either up-regulated at least two-fold or down-regulated at least two-fold. Up-regulated genes included genes (1) with a control intensity value above 100 and an Aroclor 1254 intensity value above 200, (2) with a control intensity value between 0 and 100 and a difference of at least 70 between the control and Aroclor 1254 intensity values, or (3) with the control intensity value being absent (= 0) and an Aroclor 1254 intensity value of at least 100. Down-regulated genes included genes (1) with a control intensity value above 100 and a difference of at least 70 between the control and Aroclor 1254 intensity values, or (2) with a control intensity value above 100 and the Aroclor 1254 intensity value being absent (= 0).

In the case of cDNA arrays image capture and signal quantification of hybridized PIQOR™ cDNA-arrays were done with the ScanArray3000 (GSI Lumonics) and ImaGene software Vers. 2.0 (BioDiscovery). For each spot, the local signal was measured inside of a fixed circle of 350 μm diameter and background was measured outside of the circle within specified rings 40 μm in distance to the signal and 40 μm in width. The signal and background was taken to be the average of pixels between defined low and high percentages of respective maximum intensity with percentage parameter settings for low/high being 85/97 % for signal and 10/25 % for background respectively. Local background was subtracted from signal to obtain the net signal intensity and the ratio of Cy5/Cy3. Subsequently the mean of the ratios of two corresponding spots representing the same cDNA was computed. The mean ratios were normalized to the median of all mean ratios using only spots for which the fluorescent intensity in one of the two channels was 3 times the negative control. The negative control for each array was computed as the mean of the signal intensity of two spots representing herring sperm and two spots representing spotting buffer only. Only genes displaying a 3 fold higher net signal intensity in the control or treatment sample compared to the negative control were used for further analysis.

The biological processes involving the Aroclor 1254-regulated genes were assigned by using the GOFFA library, which is a part of the public toxicogenomics software for microarray data management and analysis ArrayTrack [15]. This library provides gene ontology information using the standard vocabulary (terminology) of the Gene Ontology Consortium. Furthermore, we used the NCBI database Entrez Gene [37] and whenever necessary surveying literature in order to determine genes affected by Aroclor 1254 coded for transcription factors or regulators of cell cycle and apoptosis. Aroclor 1254-regulated transcription factors were classified according to the transcription factor classification scheme of BIOBASE GmbH [42].

### Sequence retrieval

The UCSC genome browser [38] was used to extract the promoter regions of Aroclor 1254-regulated transcription factor genes, genes involved in regulation of cell cycle and apoptosis, Aroclor 1254-regulated directly neighbored genes and promoter regions of control genes, the relative expression values of which were 1.0. Exclusively promoters of genes, which are RefSeq annotated, were extracted. The beginning of the first exon, which also comprises the 5'UTR was considered to be a tentative TSS (transcription start site) [43]. We extracted 1000 bp upstream and 100 bp downstream of TSS, respectively.

### Promoter analysis

The most widely used method for recognition of transcription factor binding sites is the application of positional weight matrices (PWMs) [39,40]. TRANSFAC^® ^Professional rel. 10.1 is the largest collection of weight matrices for eukaryotic transcription factors [41,42] (BIOBASE GmbH, Wolfenbüttel, Germany). In this database, three different weight matrices for AhR sites were stored (acc. numbers: M00139, M00235, and M00237). In cooperation with BIOBASE, we recently constructed a novel weight matrix on the basis of 25 known, experimentally verified, genomic AhR binding sites (TRANSFAC^® ^acc. number: M00778, id: V$AHR_Q5) [17]. This matrix was used for searching potential AhR sites in the different promoter sequence sets extracted (promoter sequences of Aroclor 1254-regulated transcription factors, Aroclor 1254-regulated genes involved in regulation of cell cycle and apoptosis, and control genes which are not influenced by Aroclor 1254 at all). For this search, we employed the MATCH™ algorithm calculating scores for the matches by applying the so-called information vector [44]. The cut-off values for matrix similarity (matrice binding sequence: 11 bp) used were *q *> *q*_*cut*-*off *_= 0.96 and *q *> *q*_*cut*-*off *_= 0.98 (the cut-off values for core similarity were always set to 1.00/core binding sequence: GTGCG). The matrix similarity cut-off is a score that describes the quality of a match between a matrix and an arbitrary part of the input sequences. In addition, only those matches, which score higher than or equal to the matrix similaritythreshold appear in the output. The total number of AhR matches within one sequence set was divided by the number of genes of one set. The ratio of the control promoter sequences was set to 1 and the other ratios were compared to this fold occurrence. In order to analyze the promoters of directly neighbored genes the MATCH™ algorithm was used, as well, applying the following matrices: ATF3 (TRANSFAC^® ^acc. number: M00513), v-Jun (M00036), HOXB7 (M00998), SP2 (M00933), PBX1 (M00998, M01017), MAF/NRF1 (M00983), NF-kappaB (M00208, M00774, M00054, M00194), PPARG2 (M00512, M00515), SOX18 (M01014) and BHLHB2 (M00997, M01034). The cut-off values used for the NF-kappaB and the PPARG2 matrices were default-cut-offs for core and matrice similarity, respectively. For all other matrices the cut-off value *q *> *q*_*cut*-*off *_= 1.00 for core similarity and the cut-off value *q *> *q*_*cut*-*off *_= 0.98 for matrix similarity were used. In order to define a common framework, we analyzed the promoter sequences for transcription factor binding sites with the programs MatInspector Professional and FrameWorker (Genomatix Software, Munich, Germany [45]). The computational approaches were based on the MatInspector program[39] using the selected matrix library (vertebrate section)and optimized thresholds.

### Analysis of chromosomal localization

From the list of all 910 Aroclor 1254-regulated genes a list of 857 genes was created, the chromosomal position of which are known and which possess a RefSeq accession number. The start positions of transcription of all RefSeq transcripts of the human genome (= 27400) were downloaded from the NCBI genome browser MapViewer [46]. Out of this dataset 100 random lists possessing 857 RefSeqs, respectively, were created. Distribution of both Aroclor 1254-regulated and all as yet mapped RefSeq transcripts was subsequently visualized in Microsoft Excel 2000 (Figures 3, 4 and 5). The numbers of pairs of genes for each of the lists that were located on the same chromosome within a distance of >0–50, >50–100 and >100–200 (window sizes) were calculated. The numbers obtained from the Aroclor 1254-regulated gene list were compared with the means obtained from the 100 random lists. To determine if the observed (Aroclor 1254-regulated) numbers of adjacent pairs were significantly different from those of expected adjacent pairs (100 random lists), the binominal distribution, given by the formula

B(k|p,n)=(nk)pkqn−k
 MathType@MTEF@5@5@+=feaafiart1ev1aaatCvAUfKttLearuWrP9MDH5MBPbIqV92AaeXatLxBI9gBaebbnrfifHhDYfgasaacH8akY=wiFfYdH8Gipec8Eeeu0xXdbba9frFj0=OqFfea0dXdd9vqai=hGuQ8kuc9pgc9s8qqaq=dirpe0xb9q8qiLsFr0=vr0=vr0dc8meaabaqaciaacaGaaeqabaqabeGadaaakeaacqWGcbGqcqGGOaakcqWGRbWAcqGG8baFcqWGWbaCcqGGSaalcqWGUbGBcqGGPaqkcqGH9aqpdaqadiqaauaabeqaceaaaeaacqWGUbGBaeaacqWGRbWAaaaacaGLOaGaayzkaaGaemiCaa3aaWbaaSqabeaacqWGRbWAaaGccqWGXbqCdaahaaWcbeqaaiabd6gaUjabgkHiTiabdUgaRbaaaaa@43A2@

was used. In this formula *n *is the total number of RefSeq transcripts in the gene lists (= 857), *p *is the probability of a gene pair to be present in a single window size (expected number of gene pairs in a single window size/834), *k *is the observed number of gene pairs in a single window size and *q *= 1-*p*.

## Authors' contributions

SR was responsible for the bioinformatical analysis of the study. JB initiated the study, and was responsible for the experimental part. Both authors drafted the manuscript.

## Supplementary Material

Additional File 2 **Aroclor 1254-regulated transcription factors and genes involved in regulation of cell cycle and apoptosis**. In these tables Aroclor 1254-regulated transcription factors and genes involved in regulation of cell cycle and apoptosis are listed including their Entrez Gene identifier, their chromosomal localization and the numbers of AhR sites identified in their promoters. Click here for file

Additional File 3 **Control genes**. In this table genes are listed which were not regulated by Aroclor 1254 at all, including their RefSeq identifier and the numbers of AhR sites identified in their promoters. They have been randomly selected. Click here for file

Additional File 1 **Genomic distribution of Aroclor 1254-regulated genes among the chromosomes**. This diagram shows the genomic distribution of Aroclor 1254-regulated genes among the chromosomes in comparison to the genomic distribution of all mapped genes among the chromosomes. The relative gene distribution is given on the ordinate the assignment to the respective chromosome is given on the abscissa. In case the ratio of Aroclor 1254-regulated genes on one single chromosome to all Aroclor 1254-regulated genes and the ratio of all genes mapped so far on one single chromosome to all genes mapped so far on all chromosomes is identical, the relative gene distribution is 1. Click here for file

Additional File 4 **Detailed description of 37 pairs of Aroclor 1254-regulated genes which are directly neighbored**. In this table the 37 pairs of Aroclor 1254-regulated genes which are directly neighbored are listed including their RefSeq accession numbers and whether they were induced (+) or repressed (-) by Aroclor 1254. Furthermore, the numbers of AhR binding sites in their promoters are depicted, as well as their start sites of transcription (TSS) and the distance of the start sites of the genes of one pair, respectively. Click here for file

## Figures and Tables

**Table 1:** Fold occurrence of AhR binding sites in the promoters of Aroclor 1254-regulated genes. The occurrence value of AhR binding sites in the promoters of control genes is set to 1.

**Transcription factors and co-regulators of transcription (52)**		
Control genes	Aroclor 1254-induced	Aroclor 1254-repressed
	Matrix similarity cut-off 0.96	
1	2.62	2.78
	Matrix similarity cut-off 0.98	
1	4.28	6.87

**Genes involved in regulation of cell cycle (29)**		

Control genes	Aroclor 1254-induced	Aroclor 1254-repressed
	Matrix similarity cut-off 0.96	
1	2.38	2.44
	Matrix similarity cut-off 0.98	
1	6.43	3.99

**Genes involved in apoptosis (18)**		

Control genes	Aroclor 1254-induced	Aroclor 1254-repressed
	Matrix similarity cut-off 0.96	
1	0.42	2.67
	Matrix similarity cut-off 0.98	
1	1.87	5.99

**Table 2:** Definition of a common framework consisting of different transcription factor binding sites by analyzing 32 promoters of Aroclor 1254-regulated genes (transcription factors and genes involved in cell cycle regulation and apoptosis) using the Genomatix software FrameWorker. Only these promoters were applied, which possess at least one AhR binding site in its promoter, in order to identify candidate transcription factors which might cooperate with the AhR and in order to identify a common framework for promoters regulated by the AhR.

**AROCLOR 1254-INDUCED GENES (14 SEQUENCES)**		**AROCLOR 1254-REPRESSED GENES (18 SEQUENCES)**	
**100%**	**90%**	**100%**	**90%**

AhR		AhR	
ETS		ETS	
SP1		SP1	
		E2F	
	CREB		CREB
	EGR		EGR
	ZBP		ZBP
	NF-kB		NF-kB
	NKXH		NKXH
			MAZ
			HOX
			WHZ
	ECAT		
	TBP		

Binding sites of transcription factors which were identified in Aroclor 1254-induced promoters are marked in red, whereas binding sites of transcription factors which were identified in Aroclor 1254-repressed promoters are marked in blue. Binding sites of transcription factors which were present in the corresponding frequency (100 or 90%) in only induced or repressed promoters are underlined. These transcription factor binding sites discriminate induced from repressed promoters and vice versa. ETS: ETS transcription factors are known to regulate cellular growth and differentiation. SP1: SP1 transcription factors are ubiquitous and bind to GC-rich sequences in a large number of promoters. They can modulate transcription of specific genes through interactions with cell type- or stage-specific transcription factors. E2F: The E2F family plays a crucial role in the control of cell cycle. CREB: cyclic AMP (cAMP) response element-binding protein is a ubiquitous transcription factor that functions in glucose homeostasis and stimulates the expression of numerous genes in response to growth factors, hormones, neurotransmitters, ion fluxes and stress signals. EGR: EGR transcription factors are known to activate genes which are involved in differentiation and mitogenesis. ZBP: ZBP transcription factors are known to activate genes which are involved in cell growth arrest and cell death. NF-kB: cytokine- or stress-activated and stimulates the expression of genes involved in a wide variety of biological functions. NKXH: NKX homeodomain factors play critical roles in regulating tissue-specific gene expression essential for tissue differentiation. MAZ: Myc-associated zinc fingers are ubiquitously expressed and can exert positive and negative control over the expression of target genes. HOX: The homeobox genes encode a highly conserved family of transcription factors that play an important role in morphogenesis. WHZ: Winged helix protein, involved in hair keratinization and thymus epithelium differentiation. ECAT: Enhancer CCAAT binding factors. A trimeric complex (nuclear factors YA-C (Y-box binding factors)), forming a highly conserved, ubiquitously expressed transcription factor that binds with high specificity to CCAAT motifs in the promoter regions in a variety of genes. TBP: TATA-binding protein factor binds directly to the promoter and nucleates the assembly of a transcriptionally competent preinitiation complex.

**Table 3:** Number of genes found in different window sizes: Comparison of the Aroclor 1254-regulated gene list (857 genes) with the mean of 100 randomized gene lists (857 genes, respectively).

**WINDOW SIZES ANALYSED [KBP]**	**100 RANDOMIZED GENE LISTS (857) (MEAN OF THE EXPECTED NUMBER OF GENES)**	**STANDARD DEVIATION**	**EXPECTED PROBABILITY [%]**	**AROCLOR 1254 REGULATED GENES (857) (OBSERVED NUMBER OF GENES)**	**SIGNIFICANCE (P VALUE)**
50	30.01	5.5496	3.60	54	0.00001712
100	27.15	5.5621	3.26	33	0.03838451
200	44.9	7.0231	5.38	47	0.05681548

For each window size, the expected number of genes is shown along with the observed number of genes. The expected numbers of genes arise from the mean of 100 randomized gene lists comprising the same number of genes than the Aroclor 1254-regulated gene list. The expected probability [%] describes the number of genes as percentage for a given window size. Here, all genes from the randomized lists (857) are set to 100 %. Further the p value is shown for obtaining such a result by chance using the binominal distribution.

**Table 4:** Promoter analysis of 37 pairs of Aroclor 1254-regulated genes which are directly neighbored.

**FIRST GENE (REFSEQ ACCESSION NUMBER)**	**FIRST GENE (AHR BINDING SITES)**	**FIRST GENE (BINDING SITES OF OTHER TFS)**	**SECOND GENE (REFSEQ ACCESSION NUMBER)**	**SECOND GENE (AHR BINDING SITES)**	**SECOND GENE (BINDING SITES OF OTHER TFS)**
NM_003928	-	-	NM_015582	n.d.*	n.d.*
NM_005334	-	NFKB	NM_003492	1	ATF3, SP2
NM_001643	-	PPARG2	NM_032174	1	-
NM_021642	1	-	NM_002155	-	NFKB
NM_005807	-	-	NM_003292	-	SP2
NM_006623	-	-	NM_005518	-	PPARG2
NM_002061	1	SP2	NM_000350	-	-
NM_144631	2	RFXANK	NM_002707	1	PPARG2
NM_005911	-	SP2, PPARG2	NM_000821	1	PPARG2
NM_016467	-	PPARG2	NM_000534	-	NFKB
NM_022173	1	-	NM_016297	1	RFXANK, SP2
NM_006590	1	-	NM_000542	-	NRF1, PPARG2
NM_005141	-	-	NM_000508	-	-
NM_000508	-	-	NM_000509	-	-
NM_007104	-	-	NM_003214	1	SP2
NM_138446	1	-	NM_006547	-	BHLHB2
NM_003235	-	-	NM_006748	-	-
NM_001261	-	NFKB	NM_004957	-	PBX1, SP2
NM_005564	-	NFKB	NM_024112	-	SP2
NM_005489	-	SP2	NM_001261	-	-
NM_004376	-	NFKB	NM_015960	-	PPARG2
NM_005271	5	BHLHB2	NM_019054	3	-
NM_001572	-	SP2, NFKB	NM_021924	1	SP2
NM_052854	-	SP2	NM_003646	-	SP2, PPARG2
NM_006169	-	RFXANK	NM_019021	1	PPARG2
NM_021640	-	-	NM_015665	-	NFKB
NM_003348	1	-	NM_014050	-	-
NM_002937	-	PPARG2	NM_001145	-	-
NM_006599	-	NFKB, BHLHB2	NM_000903	1	NRF1
NM_003562	1	RFXANK	NM_015528	1	NFKB, RFXANK
NM_002544	1	-	NM_006495	-	-
NM_018952	-	NFKB, RFXANK	NM_004502	2	RFXANK, BHLHB2
NM_007292	2	NFKB, BHLHB2	NM_001258	-	PPARG2
NM_005359	-	-	NM_016626	1	SP2
NM_032377	-	RFXANK	NM_001611	2	BHLHB2, PPARG2
NM_018270	2	-	NM_007346	-	RFXANK, BHLHB2
NM_002626	2	SP2	NM_004928	1	SP2

* Promoter sequence was not available

**ATF3**: activating transcription factor 3, function unknown; **BHLHB2**: basic helix-loop-helix domain containing, class B2, anti-apoptosis, oncogenesis, hypoxia-induced; **NFKB1**: Nuclear Factor kappa B1, cytokine or stress-activated; **NRF1**: nuclear respiratory factor 1, cell growth, development; **PPARG2**: Peroxisome proliferator-activated receptor gamma, adipocyte differentiation, obesity, diabetes and cancer; **RFXANK**: regulatory factor X-associated ankyrin-containing protein, controls the cell type specificity and inducibility of MHC class II gene expression; **SP2**: Sp2 transcription factor, binds to GC box promoters elements.
