# Supplementary material for: Transcriptome profiling of human hepatocytes treated with Aroclor 1254 reveals transcription factor regulatory networks and clusters of regulated genes
Source: BMC Genomics. 2006 Aug 26;7:217. doi: 10.1186/1471-2164-7-217 (PMC1590027; doi:10.1186/1471-2164-7-217)
Supplement: Additional File 2 — Aroclor 1254-regulated transcription factors and genes involved in regulation of cell cycle and apoptosis. In these tables Aroclor 1254-regulated transcription factors and genes involved in regulation of cell cycle and apoptosis are listed including their Entrez Gene identifier, their chromosomal localization and the numbers of AhR sites identified in their promoters. [file 1471-2164-7-217-S2.doc]

| **Transcription factors / induced** | | |  |  |  |  |
| --- | --- | --- | --- | --- | --- | --- |
| Gene symbol | Classification | Function | Signal ratio | Entrez  Gene ID | AhR matrix  matches in  the promoter  (q_cut-off_=0.96) | AhR matrix  matches in  the promoter  (q_cut-off_=0.98) |
|  | **Basic Domains** |  |  |  |  |  |
| DSIPI | Helix-loop-helix factor (bHLH) | anti-apoptosis | 2.46 | 1831 | - | - |
| HEY1 | Helix-loop-helix factor (bHLH) | cell-fate determination | 2.65 | 23462 | 1 | - |
| BHLHB2 | Helix-loop-helix factor (bHLH) | anti-apoptosis, oncogenesis, hypoxia-induced | 64.75 | 8553 | - | - |
| ATF3 (long) | Helix-loop-helix factor (bHLH) | unknown | 2.82 | 467 | - | - |
| JUN | Leucine zipper factor (bZIP) | oncogene | 3.22 | 3725 | - | - |
| OASIS | Leucine zipper factor (bZIP) | unknown | 2.33 | 90993 | - | - |
| CREB3 | Leucine zipper factor (bZIP) | unknown | 2.16 | 10488 | 2 | 1 |
|  | **Beta-Scaffold Factors with Minor Groove Contacts** |  |  |  |  |  |
| SOX18 | HMG | embryonic development, cell-fate determination | 11.43 | 54345 | - | - |
| MEF2C | MADS box | development, morphogenesis | 2.71 | 4208 | 1 | - |
| NRF1 | MADS box | cell growth, development | Induced* | 4899 | 3 | 2 |
| NFAT5 | RHR (Rel homology region) | osmotic response | 19.67 | 10725 | - | - |
| NFKB1 | RHR (Rel homology region) | cytokine or stress-activated | 11.19 | 4790 | 1 | - |
| NFATC3 | RHR (Rel homology region) | induction of cytokine gene expression | 2.59 | 4775 | - | - |
|  | **Helix-turn helix** |  |  |  |  |  |
| CHES1 | Fork head (winged helix) | DNA damage-inducible cell cycle arrests | 2.03 | 1112 | 1 | 1 |
| HOXB7 | Homeo domain / Homeo domain only | morphogenesis, tumor progression | 3.86 | 3217 | 2 | 1 |
| HOXB9 | Homeo domain / Homeo domain only | morphogenesis | 2.77 | 3219 | - | - |
| HOXB6 | Homeo domain / Homeo domain only | morphogenesis, development | 2.18 | 3216 | - | - |
| ZFHX1B | Homeo domain / Homeo domain plus zinc finger motifes | develpoment, activation of cancer invasion | 6.85 | 9839 | 1 | - |
| TEAD3 | TEA domain | promotes maternal breast development during pregnancy | 14.25 | 7005 | 1 | - |
|  | **Zink-coordinating DNA-binding domains** |  |  |  |  |  |
| C20orf104 | Zink-coordinating DNA-binding domains | hepatocellular carcinoma-associated antigen 58 | 2.5 | 51230 | - | - |
| TRIM16 | Zink-coordinating DNA-binding domains (B box) | estrogen and anti-estrogen regulated, cell growth, differentiation | 19.2 | 10626 | - | - |
| SP2 | Cys2His2 zinc finger domain | binds to GC box promoters elements | 2.46 | 6668 | - | - |
| KLF13 | Cys2His2 zinc finger domain | represses transcription in competition with the activator SP1 | 2.46 | 51621 | 3 | 1 |
| ZFP36L1 | Cys2His2 zinc finger domain | transcriptional regulation in response to growth factors | 3.68 | 677 | 1 | 1 |
| KLF12 | Cys2His2 zinc finger domain | development, carcinogenesis | Induced* | 11278 | 2 | - |
| PPARG | Cys4 zink finger of nuclear receptor type | adipocyte differentiation, obesity, diabetes and cancer | 2.99 | 5468 | - | - |
|  | **No classification** |  |  |  |  |  |
| UBN1 | No classification | ubiquitous, expressed in tumors and cancer cell lines | 2.71 | 29855 | 1 | - |
| **Co-regulator of transcription / induced** | | |  |  |  |  |
| CITED1 | co-regulator of transcription | melanocyte-specific gene, associated with pigmentation | 5.13 | 4435 | 2 | 1 |
| **Genes involved in regulation of cell cycle / induced** | | |  |  |  |  |
| Gene symbol | Description | | Signal ratio | Entrez  Gene ID | AhR matrix  matches in  the promoter  (q_cut-off_=0.96) | AhR matrix  matches in  the promoter  (q_cut-off_=0.98) |
| CCNT2 | cyclin T2 isoform b | | 7,17 | 905 | 2 | 1 |
| ANAPC5 | anaphase-promoting complex subunit 5 | | 6,19 | 51433 | - | - |
| BANP | BTG3 associated nuclear protein, isoform a | | 5,65 | 54971 | 2 | - |
| CCRK | cell cycle related kinase | | 5,47 | 23552 | 1 | 1 |
| CDKN1A | cyclin-dependent kinase inhibitor 1A (p21, Cip1) transcript variant 2 | | 5,46 | 1026 | 1 | 1 |
| CDK9 | cyclin-dependent kinase 9 (CDC2-related kinase) | | 3,23 | 1025 | - | - |
| SKB1 | SKB1 homolog (S. pombe) | | 3,1 | 10419 | - | - |
| CDC2L1 | cell division cycle 2-like 1, isoform 4 | | 2,74 | 984 | 1 | 1 |
| NEK6 | putative serine-threonine protein kinase, NIMA (never in mitosis gene a)-related kinase 6 (NEK6) | | 2,37 | 10783 | 2 | 1 |
| CPR2 | cell cycle progression 2 protein | | 2,06 | 9238 | - | - |
| CDC2L2 | cell division cycle 2-like 2, isoform 2, transcript variant 6 | | 2,05 | 985 | 1 | 1 |
| MCRS1 | microspherule protein 1 | | Induced* | 10445 | - | - |
| CHFR | checkpoint with forkhead and ring finger domains | | Induced* | 55743 | - | - |
| UBE2V1 | ubiquitin-conjugating enzyme E2 variant 1, isoform b | | Induced* | 7335 | - | - |
| **Genes involved in apoptosis / induced** | | |  |  |  |  |
| PLAGL1 | pleiomorphic adenoma gene-like 1 isoform 2 | | 4,84 | 5325 | - | - |
| BMF | bcl-2 modifying factor | | 4,35 | 90427 | - | - |
| TUCAN | tumor up-regulated CARD-containing antagonist of caspase nine | | 2,85 | 22900 | - | - |
| CRADD | CASP2 and RIPK1 domain containing adaptor with death domain | | 2,28 | 8738 | - | - |
| NOL3 | nucleolar protein 3 (apoptosis repressor with CARD domain) | | 2,04 | 8996 | - | - |
| LIMS1 | LIM and senescent cell antigen-like domains 1 | | 2,11 | 3987 | 1 | 1 |
| DAPK3 | death-associated protein kinase 3 | | Induced* | 1613 | - | - |
| PCBP4 | poly(rC)-binding protein 4, isoform c, transcript variant 4 | | Induced* | 57060 | - | - |

| **Transcription factors / repressed** | | | | | | | | | |
| --- | --- | --- | --- | --- | --- | --- | --- | --- | --- |
| Gene symbol | Classification | | Function | Signal ratio | | Entrez Gene ID | AhR matrix matches in  the promoter  (q_cut-off_=0.96) | | AhR matrix  matches in  the promoter  (q_cut-off_=0.98) |
|  | Basic Domains | |  |  | |  |  | |  |
| WBSCR14 | Helix-loop-helix / Leucine zipper factor (bHLH-ZIP) | | Formularbeginn  glucose metabolism and fatty acids synthesis | 0.36 | | 51085 | 1 | | 1 |
| NPAS2 | Helix-loop-helix / Leucine zipper factor (bHLH-ZIP) | | memory, molecular clock | Reduced* | | 4862 | 1 | | 1 |
| ATF3 (delta zip) | Leucine zipper factor (bZIP) | | unknown | 0.16 | | 467 | - | | - |
| HBP1 | Leucine zipper factor (bZIP) | | cell cycle inhibitor, tumor suppressor | 0.25 | | 26959 | - | | - |
|  | Helix-turn helix | |  |  | |  |  | |  |
| ID2 | Homeo domain / Homeo domain only | | Formularbeginn  negative regulation of cell differentiation | 0.28 | | 3398 | 2 | | - |
| PBX1 | Homeo domain / Homeo domain only | | development | Reduced* | | 5087 | - | | - |
| MLLT6 | Homeo domain / Homeo domain plus zinc finger motifes | | growth promoting oncogenic | 0.38 | | 4302 | 1 | | - |
| PAX6 (isoform a) | Paired box / Paired plus homeo domain | | development | 0.43 | | 5080 | 1 | | 1 |
| PAX6 (isoform b) | Paired box / Paired plus homeo domain | | development | 0.44 | | 5080 | 1 | | 1 |
| IRF7 | Tryptophan clusters | | immune response | 0.10 | | 3665 | - | | - |
|  | Zink-coordinating DNA-binding domains | |  |  | |  |  | |  |
| REST | Cys2His2 zinc finger domain | | Formularbeginn  repression of neuronal genes in non-neuronal tissues | 0.03 | | 5978 | 3 | | 1 |
| MBD1 | Cys2His2 zinc finger domain | | repression of transcription from methylated gene promoters | 0.12 | | 4152 | 1 | | 1 |
| RBAK | Cys2His2 zinc finger domain | | Formularbeginn  transcriptional repressor of cell cycle genes | 0.35 | | 57786 | 2 | | 1 |
| SCAND2 | Cys2His2 zinc finger domain | | unknown | 0.45 | | 54581 | 1 | | 1 |
| CBFA2T1 | Cys6 cysteine-zinc cluster | | Formularbeginn  oncoprotein | 0.42 | | 862 | - | | - |
| CGR19 | Zinc fingers of alternating composition | | regulation of cell cycle | 0.25 | | 10668 | 2 | | 2 |
| PML | Zinc fingers of alternating composition | | Formularbeginn  tumor suppressor, regulates the p53 response to oncogenic signals | 0.25 | | 5371 | - | | - |
| ZNF144 | Zinc fingers of alternating composition | | Formularbeginn  transcriptional repression of genes involved in cell cycles, and tumorigenesis | 0.46 | | 7703 | 1 | | - |
|  | No classification | |  |  | |  |  | |  |
| TAF2 | No classification | | serves as the scaffold for assembly of the basal transcription complex | 0.45 | | 6873 | 2 | | 1 |
| **Co-regulator of transcription / repressed** | | | | | | | | | |
| RFXANK | co-regulator of transcription | | Formularbeginn  controls the cell type specificity and inducibility of MHC class II gene expression | 0.12 | | 8625 | | - | - |
| CNOT2 | co-regulator of transcription | | cell life, proliferation/growth | 0.28 | | 4848 | | - | - |
| AF5Q31 | co-regulator of transcription | | Formularbeginn  involved in acute lymphoblastic leukemia | 0.35 | | 27125 | | 1 | - |
| MADH4 | co-regulator of transcription | | Formularbeginn  apoptosis, tumour suppressor | Reduced* | | 4089 | | - | - |
| HCFC1 | co-regulator of transcription | | possible role in cell proliferation | 0.44 | | 3054 | | - | - |
| **Genes involved in regulation of cell cycle / repressed** | | | |  |  | |  | |  |
| Gene symbol | | Description | | Signal ratio | Entrez  Gene ID | | AhR matrix  matches in  the promoter  (q_cut-off_=0.96) | | AhR matrix  matches in  the promoter  (q_cut-off_=0.98) |
| HEF1 | | enhancer of filamentation 1 (cas-like docking; Crk-associated substrate related) | | 0,15 | 10543 | | 1 | | - |
| NIN | | ninein (GSK3B interacting protein) | | 0,17 | 51199 | | - | | - |
| LGALS1 | | lectin, galactoside-binding, soluble, 1 (galectin 1) | | 0,17 | 3956 | | 1 | | 1 |
| ANAPC11 | | APC11 anaphase promoting complex subunit 11 homolog (yeast) | | 0,20 | 51529 | | 3 | | 1 |
| CENPF | | centromere protein F (350/400kD, mitosin) | | 0,24 | 1063 | | - | | - |
| MTCP1 | | mature T-cell proliferation 1 | | 0,26 | 4515 | | 1 | | - |
| RBBP6 | | retinoblastoma-binding protein 6 | | 0,33 | 5930 | | 1 | | 1 |
| BTG1 | | B-cell translocation protein 1, anti-proliferative (BTG1) | | 0,45 | 694 | | 1 | | - |
| SH3BP4 | | SH3-domain binding protein 4 | | 0,47 | 23677 | | 2 | | - |
| CDC26 | | CDC26 subunit of anaphase promoting complex | | 0,48 | 246184 | | | - | - |
| CDC25A | | cell division cycle 25A | | 0,48 | 993 | | | - | - |
| EMP3 | | epithelial membrane protein 3 | | 0,50 | 2014 | | | - | - |
| CDK3 | | cyclin-dependent kinase 3 | | Reduced* | 1018 | | | - | - |
| MAD2L1 | | MAD2-like 1, MAD2 mitotic arrest deficient-like 1 (yeast) | | Reduced* | 4085 | | | - | - |
| STIM1 | | stromal interaction molecule 1 | | Reduced* | 6786 | | | 1 | 1 |
| **Genes involved in apoptosis / repressed** | | | | | | | | | |
| SON | | SON DNA-binding protein, isoform C | | 0,07 | 6651 | | | - | - |
| CIDEB | | cell death-inducing DFFA-like effector b | | 0,17 | 27141 | | | - | - |
| TNFSF10 | | tumor necrosis factor (ligand) superfamily, member 10 | | 0,18 | 8743 | | | - | - |
| CARD10 | | caspase recruitment domain protein 10 | | 0,21 | 29775 | | | 2 | 1 |
| NTRK1 | | neurotrophic tyrosine kinase, receptor, type 1 | | 0,23 | 4914 | | | 1 | - |
| TNFSF12 | | tumor necrosis factor (ligand) superfamily, member 12 | | 0,34 | 8742 | | | 2 | 1 |
| NALP2 | | NALP2 protein | | 0,35 | 55655 | | | 1 | 1 |
| TIA1 | | TIA1 protein, isoform 2, cytotoxic granule-associated RNA binding protein | | 0,37 | 7072 | | | 1 | 1 |
| BM040 | | uncharacterized bone marrow protein BM040 | | 0,39 | 55840 | | | 1 | - |
| BBP | | beta-amyloid binding protein precursor | | 0,42 | 83941 | | | - | - |

* Induced = control intensity value was absent and Aroclor 1254 intensity value was above 100

* Reduced = control intensity value was above 100 and Aroclor 1254 intensity value was absent
