# Supplementary material for: Transcriptome profiling of human hepatocytes treated with Aroclor 1254 reveals transcription factor regulatory networks and clusters of regulated genes
Source: BMC Genomics. 2006 Aug 26;7:217. doi: 10.1186/1471-2164-7-217 (PMC1590027; doi:10.1186/1471-2164-7-217)
Supplement: Additional File 3 — Control genes. In this table genes are listed which were not regulated by Aroclor 1254 at all, including their RefSeq identifier and the numbers of AhR sites identified in their promoters. They have been randomly selected. [file 1471-2164-7-217-S3.doc]

| Gene name | Description | RefSeq ID | AhR matrix matches in the promoter  (q_cut-off_=0.96) | AhR matrix matches in the promoter  (q_cut-off_=0.98) |
| --- | --- | --- | --- | --- |
| NBEA | BCL8B|Homo sapiens neurobeachin | NM_015678 | - | - |
| ARV1 | likely ortholog of yeast ARV1 | NM_022786 | - | - |
| OPA1 | optic atrophy 1, isoform 5 (autosomal dominant), nuclear gene encoding mitochondrial protein | NM_130834 | - | - |
| C1orf16 | chromosome 1 open reading frame 16 | NM_014837 | - | - |
| FLJ13868 | hypothetical protein FLJ13868 | NM_022744 | - | - |
| NCUBE1 | CGI-76 protein, non-canonical ubquitin conjugating enzyme 1 | NM_016021 | - | - |
| ARIH2 | ariadne homolog 2 (Drosophila) | NM_006321 | 1 | 1 |
| MAPT | microtubule-associated protein tau, isoform 3 | NM_016834 | 2 | - |
| PLP1 | proteolipid protein 1, (Pelizaeus-Merzbacher disease, spastic paraplegia 2, uncomplicated) | NM_000533 | - | - |
| TRAP150 | thyroid hormone receptor-associated protein, 150 kDa subunit | NM_005119 | - | - |
| PRKCABP | protein kinase C, alpha binding protein | NM_012407 | - | - |
| ACTN1 | actinin, alpha 1 | NM_001102 | 2 | 1 |
| PCTK2 | PCTAIRE protein kinase 2 | NM_015678 | - | - |
| PITPNB | phosphotidylinositol transfer protein, beta | NM_017656 | 1 | - |
| PLN | phospholamban | NM_012399 | - | - |
| CPZ | carboxypeptidase Z | NM_002667 | - | - |
| GRSF1 | G-rich RNA sequence binding factor 1 | NM_003652 | - | - |
| PDGFB | platelet-derived growth factor beta, isoform 1 (simian sarcoma viral (v-sis) oncogene homolog) | NM_002092 | - | - |
| MCL1 | myeloid cell leukemia sequence 1 (BCL2-related) | NM_021960 | - | - |
| FGFR2 | fibroblast growth factor receptor 2, isoform 10 (bacteria-expressed kinase, keratinocyte growth factor receptor, craniofacial dysostosis 1, Crouzon syndrome, Pfeiffer syndrome, Jackson-Weiss syndrome) | NM_023028 | 1 | - |
| GAA | acid alpha-glucosidase (Pompe disease, glycogen storage disease type II) | NM_000152 | - | - |
| ABCC1 | ATP-binding cassette, sub-family C, member 1, isoform 2 (CFTR/MRP) | NM_019862 | - | - |
| ATP6V1G1 | ATPase, H+ transporting, lysosomal, V1 subunit G isoform 1 | NM_004888 | - | - |
| OSMR | oncostatin M receptor | NM_003999 | - | - |
| CUTL1 | cut-like 1, CCAAT displacement protein (Drosophila) | NM_001913 | 1 | - |
| FLJ20399 | hypothetical protein FLJ20399 | NM_017803 | - | - |
| FLJ20445 | hypothetical protein FLJ20445 | NM_017824 | 1 | - |
| MGC10870 | hypothetical protein MGC10870 | NM_032301 | - | - |
| UGTREL1 | UDP-galactose transporter related | NM_005827 | - | - |
| FLJ20312 | hypothetical protein FLJ20312 | NM_017761 | - | - |
