# Supplementary material for: Transcriptome profiling of human hepatocytes treated with Aroclor 1254 reveals transcription factor regulatory networks and clusters of regulated genes
Source: BMC Genomics. 2006 Aug 26;7:217. doi: 10.1186/1471-2164-7-217 (PMC1590027; doi:10.1186/1471-2164-7-217)
Supplement: Additional File 4 — Detailed description of 37 pairs of Aroclor 1254-regulated genes which are directly neighbored. In this table the 37 pairs of Aroclor 1254-regulated genes which are directly neighbored are listed including their RefSeq accession numbers and whether they were induced (+) or repressed (-) by Aroclor 1254. Furthermore, the numbers of AhR binding sites in their promoters are depicted, as well as their start sites of transcription (TSS) and the distance of the start sites of the genes of one pair, respectively. [file 1471-2164-7-217-S4.doc]

| Chromosome | RefSeq  accession  numbers | Induced /  repressed | Numbers of AhR sites  in the promoter | TSS [bp] of the first gene | TSS [bp] of the second gene | Distance between TSSs  [bp] |
| --- | --- | --- | --- | --- | --- | --- |
| X | NM_003928 / NM_015582 | - / - | 0 / n.d.* | 132,872,000 | 132,890,576 | 18,576 |
| X | NM_005334 / NM_003492 | - / + | 0 / 1 | 151,680,912 | 151,706,144 | 25,232 |
| 1 | NM_001643 / NM_032174 | - / + | 0 / 1 | 158,408,880 | 158,412,640 | 3,760 |
| 1 | NM_021642 / NM_002155 | - / + | 1 / 0 | 158,744,032 | 158,763,120 | 19,088 |
| 1 | NM_005807 / NM_003292 | - / + | 0 / 0 | 183,504,976 | 183,521,952 | 16,976 |
| 1 | NM_006623 / NM_005518 | + / - | 0 / 0 | 119,600,888 | 119,637,384 | 36,496 |
| 1 | NM_002061 / NM_000350 | + / + | 1 / 0 | 93,825,816 | 93,930,192 | 104,376 |
| 2 | NM_144631 / NM_002707 | - / - | 2 / 1 | 27,574,640 | 27,578,596 | 3,956 |
| 2 | NM_005911 / NM_000821 | + / - | 0 / 1 | 85,740,840 | 85,750,744 | 9,896 |
| 2 | NM_016467 / NM_000534 | - / + | 0 / 0 | 190,837,600 | 190,851,808 | 14,208 |
| 2 | NM_022173 / NM_016297 | - / + | 1 / 1 | 70,413,352 | 70,459,824 | 46,480 |
| 2 | NM_006590 / NM_000542 | + / - | 1 / 0 | 85,817,848 | 85,860,528 | 41,128 |
| 4 | NM_005141 / NM_000508 | - / - | 0 / 0 | 156,061,792 | 156,081,904 | 20,112 |
| 4 | NM_000508 / NM_000509 | - / - | 0 / 0 | 156,081,904 | 156,102,912 | 21,008 |
| 6 | NM_007104 / NM_003214 | - / + | 0 / 1 | 35,483,052 | 35,488,284 | 5,212 |
| 7 | NM_138446 / NM_006547 | - / - | 1 / 0 | 23,081,212 | 23,092,100 | 10,888 |
| 8 | NM_003235 / NM_006748 | - / + | 0 / 0 | 133,835,792 | 134,005,560 | 169,768 |
| 9 | NM_001261 / NM_004957 | + / - | 0 / 0 | 125,924,384 | 125,941,240 | 16,856 |
| 9 | NM_005564 / NM_024112 | + / - | 0 / 0 | 126,287,760 | 126,298,680 | 10,920 |
| 9 | NM_005489 / NM_001261 | - / + | 0 / 0 | 125,877,072 | 125,924,384 | 47,736 |
| 10 | NM_004376 / NM_015960 | - / + | 0 / 0 | 101,136,192 | 101,156,592 | 20,400 |
| 10 | NM_005271 / NM_019054 | - / - | 5 / 3 | 88,474,824 | 88,519,528 | 44,712 |
| 11 | NM_001572 / NM_021924 | - / - | 0 / 1 | 602,810 | 607,510 | 4,032 |
| 11 | NM_052854 / NM_003646 | + / + | 0 / 0 | 46,263,868 | 46,333,432 | 69,564 |
| 11 | NM_006169 / NM_019021 | - / - | 0 / 1 | 113,704,824 | 113,807,448 | 126,968 |
| 12 | NM_021640 / NM_015665 | - / - | 0 / 0 | 51,979,768 | 51,987,508 | 7,740 |
| 12 | NM_003348 / NM_014050 | - / + | 1 / 0 | 92,304,552 | 92,363,736 | 59,176 |
| 14 | NM_002937 / NM_001145 | - / - | 0 / 0 | 19,142,500 | 19,151,740 | 9,240 |
| 16 | NM_006599 / NM_000903 | + / + | 0 / 1 | 69,375,184 | 69,519,496 | 144,312 |
| 17 | NM_003562 / NM_015528 | - / - | 1 / 1 | 5,041,189 | 5,044,215 | 3,026 |
| 17 | NM_002544 / NM_006495 | - / - | 1 / 0 | 29,767,156 | 29,776,356 | 9,196 |
| 17 | NM_018952 / NM_004502 | + / + | 0 / 2 | 47,147,752 | 47,159,232 | 11,480 |
| 17 | NM_007292 / NM_001258 | + / - | 2 / 0 | 74,538,784 | 74,594,544 | 55,760 |
| 18 | NM_005359 / NM_016626 | - / + | 0 / 1 | 46,808,600 | 46,952,908 | 144,308 |
| 19 | NM_032377 / NM_001611 | + / + | 0 / 2 | 11,524,861 | 11,546,476 | 21,616 |
| 20 | NM_018270 / NM_007346 | - / + | 2 / 0 | 62,154,184 | 62,162,524 | 8,340 |
| 21 | NM_002626 / NM_004928 | - / - | 2 / 1 | 44,576,172 | 44,605,028 | 28,856 |

* Promoter sequence was not available
