# Supplementary material for: Transcriptome profiling of human hepatocytes treated with Aroclor 1254 reveals transcription factor regulatory networks and clusters of regulated genes
Source: BMC Genomics. 2006 Aug 26;7:217. doi: 10.1186/1471-2164-7-217 (PMC1590027; doi:10.1186/1471-2164-7-217)
Supplement: Supplementary file 4 [file 1471-2164-7-217-S1.doc]

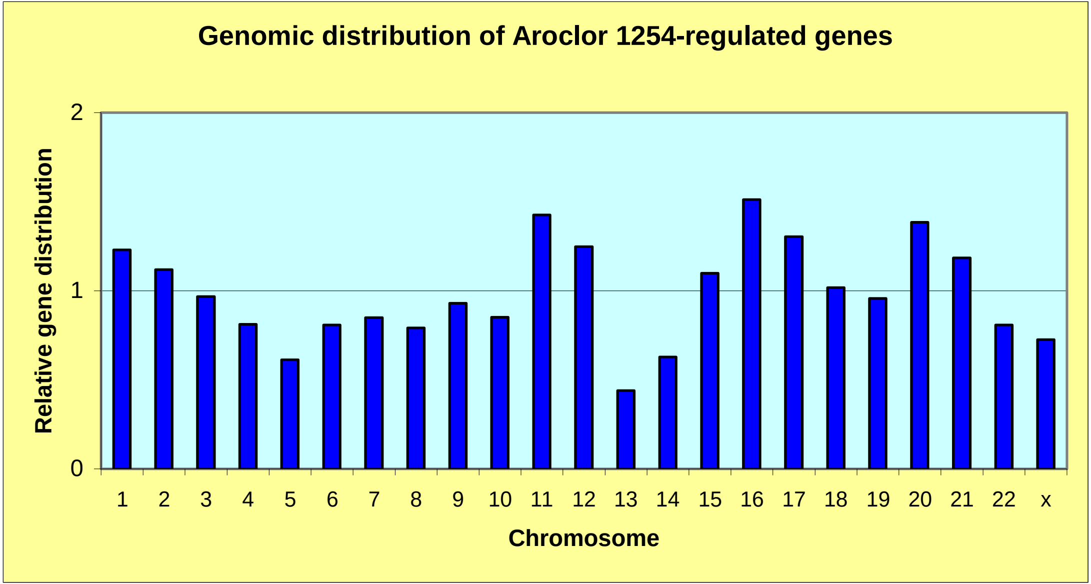
Genomic distribution of Aroclor 1254-regulated genes
Relative gene distribution
2
1
0
1 2 3 4 5 6 7 8 9 10 11 12 13 14 15 16 17 18 19 20 21 22 x
Chromosome
